# Demographic Characteristics of World Class Jamaican Sprinters

**DOI:** 10.1155/2013/670217

**Published:** 2013-12-10

**Authors:** Rachael Irving, Vilma Charlton, Errol Morrison, Aldeam Facey, Oral Buchanan

**Affiliations:** ^1^Department of Basic Medical Sciences, Faculty of Medical Sciences, University of the West Indies, Mona, Kingston 6, Jamaica; ^2^Institute of Education, University of the West Indies, Kingston 6, Jamaica; ^3^University of Technology, Kingston 7, Jamaica

## Abstract

The dominance of Jamaican sprinters in international meets remains largely unexplained. Proposed explanations include demographics and favorable physiological characteristics. The aim of this study was to analyze the demographic characteristics of world class Jamaican sprinters. Questionnaires administered to 120 members of the Jamaican national team and 125 controls elicited information on place of birth, language, ethnicity, and distance and method of travel to school. Athletes were divided into three groups based on athletic disciplines: sprint (s: 100–400 m; *n* = 80), jump and throw (j/t: jump and throw; *n* = 25) and, middle distance (md: 800–3000 m; *n* = 15). Frequency differences between groups were assessed using chi-square tests. Regional or county distribution of sprint differed from that of middle distance (*P* < 0.001) but not from that of jump and throw athletes (*P* = 0.24) and that of controls (*P* = 0.59). Sprint athletes predominately originated from the Surrey county (s = 46%, j/t = 37%, md = 17, C = 53%), whilst middle distance athletes exhibited excess from the Middlesex county (md = 60%). The language distribution of all groups showed uniformity with a predominance of English. A higher proportion of middle distance and jump and throw athletes walked to school (md = 80%, j/t = 52%, s = 10%, and C = 12%) and travelled greater distances to school. In conclusion, Jamaica's success in sprinting may be related to environmental and social factors.

## 1. Introduction

The success of Jamaicans in the sprint events during the decades of Olympic participation from 1948 to 2012 reached a crescendo at the Olympic Games in London in 2012. Jamaicans won 12 sprint medals and had a 1-2 finish in the men's 100 m final, an 1-2-3 finish in the men's 200 m final, and a gold medal in the women's 100 m final. Jamaica has three of the world's four fastest men at 100 m. Several studies have tried to explain the success of Jamaicans in the sprint events. Proposed mechanisms include favorable physiological characteristics that could be environmentally, regionally, or genetically determined [1]. Psychological programming also helps in molding sprinting talent; there is a culture of running in Jamaica with young children being actively involved in sprinting competitions [2]. Studies have compared the physiological characteristics of “black” and “white” athletes, reporting that the former have lower levels of blood and muscle lactate at a given exercise intensity [3] and a greater ability to tolerate higher fractional utilization of maximal oxygen uptake (VO_2_ max) [4]. Athletes of African descent have a higher percentage of fast-twitch muscle fibres, greater activity in the glycolytic, phosphagenic, and lactate dehydrogenase metabolic pathways, and greater rate of ventilation [1]. The limitation of extrapolating the findings of these studies is that these studies have classified groups based primarily on skin color without accounting for the fact that there are sometimes more differences within races than between; also these findings are not exclusive to athletes [5]. The influence of the compensatory sickle cell gene on oxygen transport and availability to the tissues is reported to give black athletes an advantage in sprinting [1]. It is postulated that the reduced availability coupled with the reduced myoglobin in the preponderant fast-twitch muscle fibers which are adapted for rapid energy (ATP) regeneration, all give a net outcome of muscle anatomical and biochemical advantages which proffer a superior performance [1]. Performance-related genes, biomechanics and the environment have been implicated in elite sporting performance [6, 7]; however, no study has been done that specifically looks at the demographics of Jamaica's world class sprinters.

Jamaica was selected as the model for the present study as the country's athletes have an unparalleled record of success at the international level dating as far back as 1952 when the men of the 4 × 400 meters relay team set a world record at the Olympics in Helsinski. Jamaica has a population of approximately 2.7 million people distributed in 3 counties consisting of 14 parishes. The country is English speaking. Jamaica was colonized by the English in 1634 and until 1962 it was under direct British rule. The country's population according to a United Nations report [8] consists of predominate blacks whose ancestors originated from West and West-Central Africa [9].

To our knowledge, no study has attempted to trace the ethnic or environmental background of world class Jamaican sprinters and by demographics determine the possibility that they might share a common ethnic or environmental origin. The aim of this study therefore was to determine the demographic characteristics of world class Jamaican sprinters. The findings were then compared with those of the general (nonathletic) Jamaican population to determine whether the sprinters differ in demographics from the ordinary population.

## 2. Methods

The study was approved by the University Hospital of the West Indies, Kingston, Jamaica Ethics Committee. Written informed consents were obtained from the 245 participants. The experimental procedures were in accordance with the policy statement of the American College of Sports Medicine. Participants comprised 120 elite athletes, many of whom were world and Olympic record holders, and 125 control participants. All the athletes had represented Jamaica at international games. The control participants, 125 students from the G.C. Foster College and the University of the West Indies, Mona, were intended to be representative of the general Jamaican population (C: Controls, *n* = 125). This group does not actively participate in sports at the professional or amateur level. The athletes were divided into three groups based on athletic disciplines: sprint (s: 100–400 m, *n* = 80), jump and throw (j/t: jump and throw, *n* = 25), and middle distance (md: 800–3000 m, *n* = 15). Athletes in the sprints were truly elite athletes, regularly dominating in international sprint events; many were current or former world, Olympic, and Commonwealth record holders. Although Jamaica is not usually successful internationally in middle distance events (800–3000 m), these athletes were included in the study to investigate the possibility of disproportionate number of athletes originating from a particular geographical region being the result of an abundant prominence of athletics in that region. The questionnaires used were written in English and modeled off those used in two similar demographic studies done on world class athletes from Kenya and Ethiopia [10, 11]. Questions were simple and were explained to those who could not easily understand. The questions were designed to obtain the following information. 

**Figure 1:**
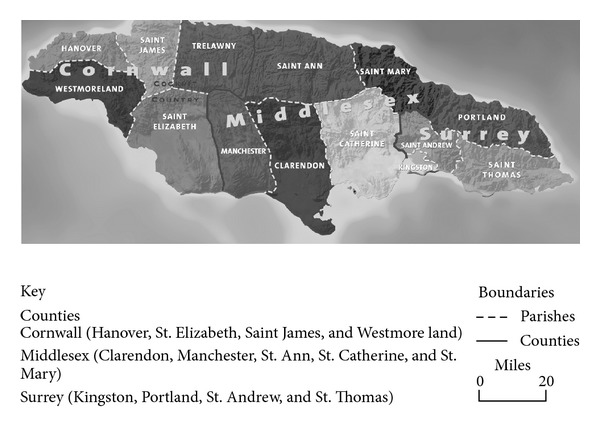
Parishes of Jamaica divided in the three counties: Cornwall, Middlesex and Surrey.

*Place of Birth*. This was classified according to the 14 parishes (Figure 1) and three counties of Jamaica [12]. The intention was to identify particular regions with a disproportionate high number of athletes in response to reports that the majority of Jamaica's most successful sprinters are from the county of Cornwall and in particular the parish of Trelawny.

*Spoken Language and That of Parents and Grandparents*. This serves to provide information on ethnicity. A common language is often indicative of common origin, and a related language or a language of the same family indicates a common origin dating further back in time [13]. At present only two languages are used by most Jamaicans: English and Patois (Creole-English).

*Mode (Walk, Run, and Transport) and Distance Travelled to School (2 Km, 2–5 Km, 5–10 Km, 10–15 Km, and >15 Km)*. This was used to access the link between distance travelled to school and running success.

## 3. Data Analysis

Contingency chi-squares using IBM SPSS Statistics 20 were performed using the Yates, correction factor in all occasions to identify frequency differences between groups given the low subject numbers in each field (place of birth, languages, ethnicity, mode, and distance travelled to school).

Individual chi-squares were then performed to identify between which groups the differences lay (place of birth: df = 12, language: df = 1, ethnicity: df = 4, distance travell to school: df = 4 and method of travelled to school: df = 2). Statistical significance was defined as *P* ≤ 0.05. The 14 parishes were collapsed into the three counties of Jamaica to allow for statistical analysis using contingency chi-squares.

## 4. Results

### 4.1. Place of Birth

**Figure 2:**
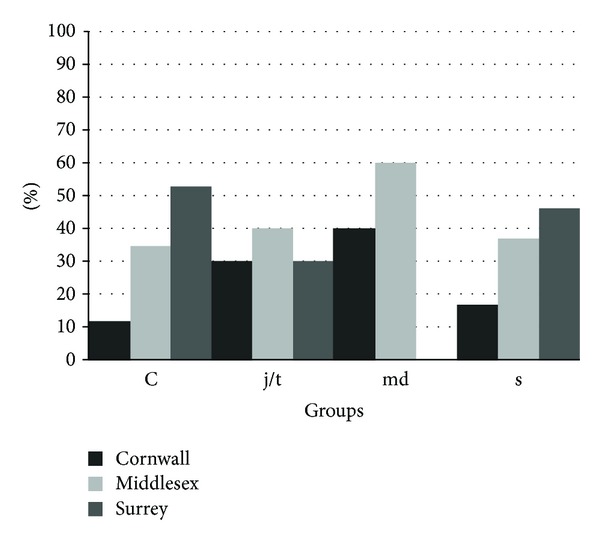
Place of birth of athletes and controls. County distribution of controls did not differ from the sprint and jump and throw athletes (*P* = 0.59 and 0.23) but differed from middle distance athletes (*P* < 0.0001). The county distribution of middle distance athletes differed significantly from that of sprint athletes (*P* < 0.001).

County or regional distribution of sprint (s) and jump and throw athletes (j/t) did not differ from that of the controls (*χ* ^2^ = 3.4, *P* = 0.59 and *χ* ^2^ = 7.5 and *P* = 0.23, resp.) but county distribution of controls differed significantly from that of middle distance athletes (*χ* ^2^ = 40 and *P* < 0.0001). The county distribution of sprint and middle distance athletes differed significantly (*χ* ^2^ = 30, *P* < 0.0001). County distribution of jump and throw athletes did not differ significantly from that of sprint athletes (*χ* ^2^ = 6.1, *P* = 0.24). Most of the sprint athletes were from the county of Surrey (46% versus 37% from Middlesex and 17% from Cornwall). The controls were mainly from Surrey (53%). There was a marked overrepresentation of middle distance athletes in the county of Middlesex (60% versus 40% from Cornwall and 0% from Surrey, see Figure 2). Only 12% of the control participants were from Cornwall. Jump and throw athletes were distributed across the three counties (Surrey: 30%, Middlesex: 40%, and Cornwall: 30%).

### 4.2. Language

The language spoken did not differ significantly (*P* > 0.05) amongst any of the groups (C versus s, C versus j/t, C versus md, s versus j/t, s versus md, and j/t versus md).

### 4.3. Mode of Travel to School

The mode of travelling to school did not differ between the controls and sprint athletes but differed slightly between the controls and jump and throw athletes (*χ* ^2^ = 0.4, *P* = 0.8 and *χ* ^2^ = 10.4, *P* < 0.05, resp.); however, there was a significant difference between the controls and middle distance athletes (*χ* ^2^ = 29.6, *P* < 0.001). A significant difference was seen between the sprint and middle distance athletes (*χ* ^2^ = 32.1, *P* < 0.0001) and between the jump and throw and middle distance athletes (*χ* ^2^ = 22.1, *P* < 0.001, see Table 1).

### 4.4. Distance Travelled to School

**Figure 3:**
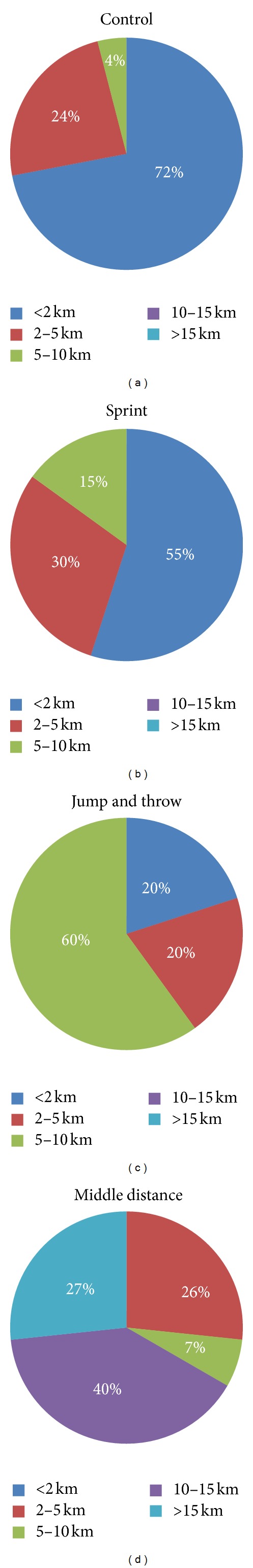
Distances travelled to school. Charts showing percentage of participants and distances traveled to school daily. The jump and throw and middle distance athletes differed significantly from the sprint athletes and controls.

The distance travelled to school did not differ between the controls and sprint athletes (*χ* ^2^ = 5.2, *P* = 0.058) but differed between the controls and the jump and throw athletes (*χ* ^2^ = 13.1, *P* > 0.001). Seventy percent of the controls Travelled 2 kilometers or less to school (see Figure 3). Fifty five percent of the sprint athletes and 20% of the jump and throw athletes travelled ≤ 2 kilometres to school. The control and the middle distance athletes differed significantly in the distance travelled to school (*χ* ^2^ = 23.4, *P* < 0.001). Forty percent (40%) of the middle distance athletes travelled between 10–15 km to school and approximately 26.7% travelled >15 km to school. All the athletic groups differed significantly in the distance travelled to school (s versus j/t: *χ* ^2^ = 14.1, *P* < 0.01; s versus md, *χ* ^2^ = 20.1, *P* < 0.001; j/t versus md: *χ* ^2^ = 15.2, *P* < 0.001).

## 5. Discussion

The study showed that Jamaican sprinters are of similar environmental and ethnic background as ordinary Jamaicans or controls. The sprint athletes were distributed across the island with 46% from Surrey, 37% from Middlesex, and 17% from Cornwall. The controls originated from across the island with 53% originating from the Surrey county. There were no differences between the sprint athletes and the controls in the distance and the mode they travelled to school. Most use a private automobile or the public bus. No population stratification was identified between controls and sprint athletes, as seen in the areas where both groups reside.

The jump and throw athletes showed a slight dominance in the county of Middlesex (40%); however, they were equally represented in Cornwall and Surrey (30% and 30%). There was a significant difference in the distance travelled to school between the sprint and jump and throw athletes (*χ* ^2^ = 14.1, *P* < 0.001). Approximately 60% of the jump and throw athletes travelled 5–10 kilometres to school and approximately 55% of the sprint athletes travelled ≤2 km to school. There was a slight difference between the jump and throw athletes and the controls in the mode travelling to school (*P* < 0.05).

The middle distance runners seemed to be of a distinct environment or county background. Most of the middle distance runners were from the county of Middlesex (60%) which consists of the parishes of Clarendon, Manchester, St. Ann, St. Catherine, and St. Mary. Many of these parishes are deep rural with unreliable means of travel. Roads are often dirty and distances between schools are much greater than those in the county of Surrey which is more developed in terms of modern amenities. Another 40% of the middle distance athletes originated from Cornwall which has more mountainous parishes than Middlesex. Middlesex however forms part of the Blue Mountain range with highest point at about 2,250 meters [14]. No middle distance athletes originated from the urban Surrey but 53% of controls were from Surrey. Surrey also forms part of the Blue Mountain range but the controls and sprinters tend to originate from the valleys in the Surrey county. Environment and not ethnicity seemed to be the factor that differentiated the middle distance runners from the sprinters as both groups mainly spoke Creole English and English. A common language is suggestive of same origin and a related language may also indicate a common origin, but one that is older [13]. The middle distance runners were over represented in Middlesex and Cornwall which consist of mountainous areas. High altitude may be benefital in distance running [7]. This finding supports the theory that the success of the Kenyans in distance running in some way may be linked to the proximity of the Rift Valley as many of Kenyans distance runners are from the high altitude, Rift Valley region. Jamaican middle distance runners seemed to be a special set from the mountainous areas. The middle distance runners travelled longer distance to school (93% travelled > 5 km to school versus 60% of the jump and throw athletes and 15% of the sprint athletes) and were more likely and jump and throw athletes to travel to school via bicycle or walking than the sprinters. The significant differences between controls and middle distance athletes in regards to place of birth, distance travelled to school, and mode of travel suggested some link between place of birth and middle distance athletic ability. The finding that the middle distance athletes seem to be clustered in the deep rural parishes or counties of Jamaica may support the hypothesis of a link between mountain and endurance. The athletes from clusters in the deep rural parishes showed more African haplotypes than the general population [2]. When this study is compared to the findings that Kenyans who walked or run to school had VO_2_ max values that are 30% higher than those who did not walk or run [10] there is an implication that childhood endurance activity might be a determinant of middle distance athletic selection.

## 6. Conclusion

The results showed that world class Jamaican sprinters have the same demographic profile as the general population. The middle distance athletes seem to have a distinct demographic profile for all variables except language.

## Figures and Tables

**Table 1:** Mode of travel by groups.

Groups	Mode of travel to school		
	Walk	Bicycle	Transport (bus, car)
Jump and throw	10 (40%)	2 (8%)	13 (52%)
Middle distance	12 (80%)	3 (20%)	0
Sprint	12 (10%)	0	108 (90%)
Control	15 (12%)	0	110 (88%)

The mode of travel to school did not differ between the controls and sprint athletes but differs slightly between the controls and jump and throw athletes (*χ* ^2^ = 0.4, *P* = 0.8 and *χ* ^2^ = 10.4 and *P* < 0.05, resp.).

There was a significant difference between the controls and middle distance athletes (*χ* ^2^ = 29.6, *P* = 0.001).

There was also a significant difference between the sprint and middle distance athletes (*χ* ^2^ = 32.1 and *P* = 0.0001) and between the jump and throw and middle distance athletes (*χ* ^2^ = 22.1, *P* = 0.001).
